# COPING WITH AMYOTROPHIC LATERAL SCLEROSIS (ALS): EXPERIENCES OF PERSONS LIVING WITH ALS AND THEIR FAMILY CAREGIVERS

**DOI:** 10.1093/geroni/igae098.0774

**Published:** 2024-12-31

**Authors:** Everlyne Ogugu, Orly Tonkikh, Jessica Famula, Heather Young, Janice Bell, Bronwyn Fields, Bjorn Oskarsson

**Affiliations:** University of California Davis, Davis, California, United States; University of California Davis, Davis, California, United States; University of California Davis, Davis, California, United States; UC Davis, Ashland, Oregon, United States; UC Davis, Davis, California, United States; Sacramento State University, Sacramento, California, United States; Mayo Clinic, Rochester, Minnesota, United States

## Abstract

Amyotrophic lateral sclerosis (ALS) is a progressive neurodegenerative disease that profoundly impacts the lives of people with the condition and their family caregivers. Little is known about the experiences of care at home among individuals with ALS and their family caregivers. This qualitative study explored these experiences in 14 in-depth interviews that included seven persons with ALS and 13 family caregivers. The findings were organized based on the Salutogenic Model of Health. The main sources of stress reported included the overwhelming nature of getting an ALS diagnosis, inadequacy of health care teams in providing emotional support and promoting shared decision-making in regard to the management of ALS, high level of illness acuity, the intense nature of caregiving involved in ALS, high cost of treatments and equipment, difficulties navigating the healthcare system, unavailability of suitable medical equipment, scarcity of paid caregivers competent in managing ALS, and lack of adequate training for family caregivers. Perspectives on positive coping included mental toughness, maintaining a positive outlook about life and the future, being proactive in seeking support to maintain function and quality of life, proactively communicating with care teams, advanced care planning, having a sense of control over ALS management plan of care, and taking a break from caregiving. Instrumental and emotional support from family, friends, community organizations, and healthcare teams, as well as sufficient insurance and financial coverage for ALS treatments, facilitated better coping. Future research and policies should focus on identifying effective multisectoral strategies for supporting people with ALS and their caregivers.

